# DNA Stability in Biodosimetry, Pharmacy and DNA Based Data‐Storage: Optimal Storage and Handling Conditions

**DOI:** 10.1002/cbic.202200391

**Published:** 2022-09-14

**Authors:** Leo Cordsmeier, Marc Benjamin Hahn

**Affiliations:** ^1^ Bundesanstalt für Materialforschung und Prüfung 12205 Berlin Germany; ^2^ Freie Universität Berlin Institut für Chemie 14195 Berlin Germany

## Abstract

DNA long‐term stability and integrity is of importance for applications in DNA based bio‐dosimetry, data‐storage, pharmaceutical quality‐control, donor insemination and DNA based functional nanomaterials. Standard protocols for these applications involve repeated freeze‐thaw cycles of the DNA, which can cause detrimental damage to the nucleobases, as well as the sugar‐phosphate backbone and therefore the whole molecule. Throughout the literature three hypotheses can be found about the underlying mechanisms occurring during freeze‐thaw cycles. It is hypothesized that DNA single‐strand breaks during freezing can be induced by mechanical stress leading to shearing of the DNA molecule, by acidic pH causing damage through depurination and beta elimination or by the presence of metal ions catalyzing oxidative damage *via* reactive oxygen species (ROS). Here we test these hypotheses under well defined conditions with plasmid DNA pUC19 in high‐purity buffer (1xPBS) at physiological salt and pH 7.4 conditions, under pH 6 and in the presence of metal ions in combination with the radical scavengers DMSO and Ectoine. The results show for the 2686 bp long plasmid DNA, that neither mechanical stress, nor pH 6 lead to degradation during repeated freeze‐thaw cycles. In contrast, the presence of metal ions (Fe^2+^) leads to degradation of DNA *via* the production of radical species.

## Graphical abstract

DNA stability is fundamental for many applications that often involve freeze‐thaw cycles, which can cause damage. It was hypothesized that the damage is related to mechanical stress, to acidic pH, or to metal ions. These hypotheses were tested. The results show that only the presence of metal ions leads to DNA damage by production of radical species during freeze‐thaw cycles.

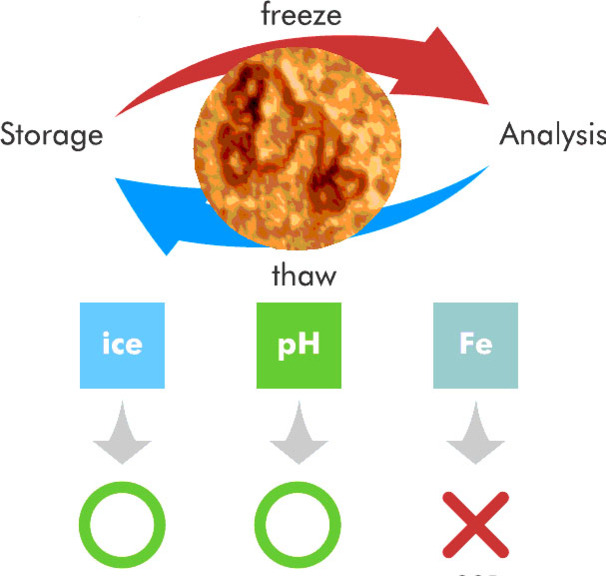

## 1 Introduction

The optimization of DNA stability under different storage and handling conditions is of importance for future applications in DNA based computation and data storage,[^1^, ^2^] the usage of plasmid DNA in pharmaceutical research, such as gene therapy and vaccine development,[^3^, ^4^, ^5^, ^6^, ^7^] novel approaches in bio dosimetry,[^8^, ^9^, ^10^] the study of effects of radio sensitisers for cancer treatment,[^11^, ^12^, ^13^] cryopreservation of human semen for donor insemination,^[14]^ and DNA origami,[^15^, ^16^, ^17^, ^18^] as well as DNA based functional nanomaterials.[^19^, ^20^, ^21^] Hereby, important aspects of DNA stability are the integrity of the DNA sugar‐phosphate backbone, as well as the correctness of the DNA base‐sequence.^[22]^ Damage to the DNA sugar‐phosphate backbone, which is responsible for the overall structure of the DNA molecule, can lead to DNA strand‐breaks. The DNA base sequence can be altered by base‐damage or base‐loss, resulting in a possible alteration of the genetic information. In a broad sense, many of these applications mentioned above, have in common, that DNA molecules with well‐defined length, structure, and base‐sequence can be stored within a predefined buffer, guaranteeing fixed DNA and salt concentrations, as well as pH. For this study we have chosen 1xPBS as the buffer since it represents physiological conditions in terms of salt concentration and pH and is often used in biochemistry and radio‐biological studies.[^23^, ^24^]

Throughout the literature, it is often mentioned that there exists a tradeoff between the higher long‐term stability of DNA stored in the frozen state at T≤‐25 °C with the related thaw‐freeze cycles when accessing it, compared to assumed faster degradation during storage in liquid at around 4 °C.[^1^, ^25^, ^26^] When working with DNA that is stored in the frozen state, each time the DNA is accessed it has to be thawed and frozen again. This is of concern as it has been shown that repeated free‐thaw cycles can prevent replication of the DNA, shorten the DNA, decrease their mechanical strength under tension, and cause base loss and DNA strand breaks.[^26^, ^27^, ^28^, ^29^] Despite the importance of DNA damage during storage or by repeated free‐thaw‐cycles, the underlying mechanisms are currently not very well understood. Throughout the literature three different explanations are given, as summarized by Brunstein.^[30]^ Thereby, damage to the DNA‐backbone is hypothesized to occur by the following pathways: When freezing the DNA ice crystals form, which exert a shearing force on the structure of the DNA and can cause the cleavage of the phosphate backbone, resulting in a single‐strand break (SSB). Another possible degradation pathway is acid catalyzed. Here, first a depurination can occur, followed by a *β*‐elimination.[^31^, ^32^, ^33^] Additionally, damage to the DNA molecule can be caused by traces of transition metals such as iron and copper in the solution.^[4]^ These metals can undergo Fenton's reactions with oxygen present in the solution to form reactive oxygen species (ROS), which then can react with various parts of DNA causing SSBs or base damage.[^3^, ^4^, ^34^] In brief, these mechanisms can be categorized as:

1. Mechanical stress leading to shearing of the DNA molecule.
2. Acidic pH causing damage by *e. g*. depurination and *β* elimination.
3. Metal ions catalyzing oxidative damage *via* ROS.

**Figure 1:**
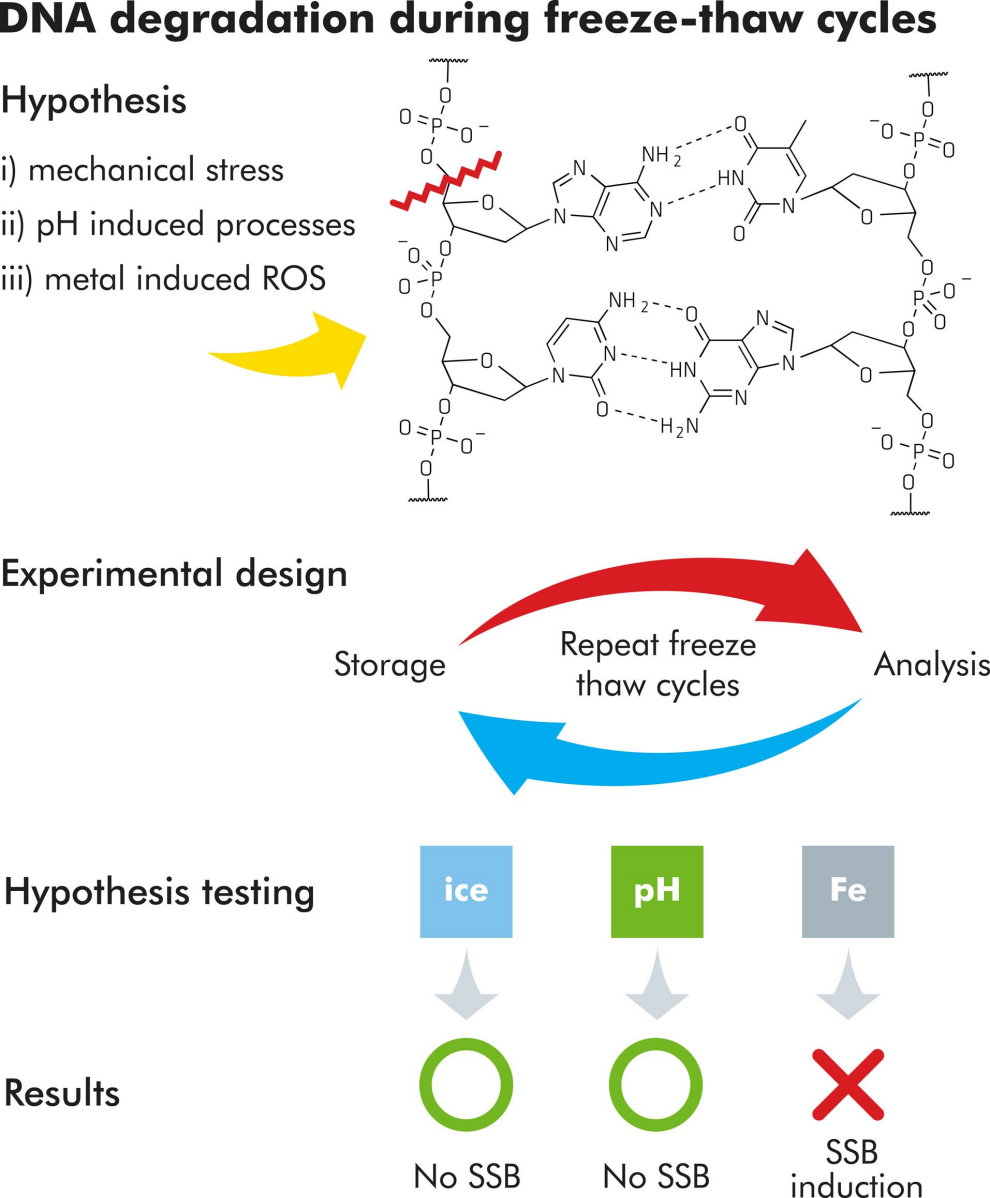
Proposed degradation mechanism during freeze‐thaw cycles and the experimental design to test the related hypotheses.

These damage mechanisms were proposed based on the prior knowledge, which was obtained from studies performed in liquid under ambient conditions at room temperature.[^30^, ^32^] However, systematic studies testing all aspects of this hypothesis for the technologically important plasmid DNA undergoing repeated free‐thaw‐cycles samples are absent from the literature. Therefore we aim to test them systematically (compare Figure 1) for a well defined system of high‐purity plasmid DNA pUC19 with 2686 base pairs under physiological conditions in 1 x PBS at pH 7.4. This plasmid DNA was chosen since it is currently being evaluated as a candidate for a future reference material with applications in pharmacy and biodosimetry.^[35]^ Additionally, experiments with respect to the influence of typical sample handling procedures and varying storage conditions were performed. Especially since it is an often discussed point that DNA can be degraded by typical handling procedures, such as mechanical stress introduced by pipetting or vortexing.^[36]^ Furthermore long‐term monitoring of plasmid DNA stability stored at T≤−25 °C was performed over a period of 20 month for an extensive set of samples. Hereby the change in the relative amount of supercoiled plasmid DNA over time was evaluated, which is directly related to the induction of DNA single‐strand breaks and therefore to the structural integrity of the DNA molecule itself.^[23]^

## 2 Results and discussion

In the present study we have performed repeated freeze‐thaw cycles with plasmid DNA to test different hypotheses about the underlying physical and chemical damage mechanisms during the freeze‐thaw processes. The first mechanism proposed for DNA strand‐break induction was (1.) shearing of DNA molecules by mechanical stress during the formation of ice crystals. The second proposed mechanisms was (2.) DNA strand breakage *via* processes induced by acidic pH. As a third mechanism (3.), oxidative damage by ROS was proposed, which can be catalyzed by the presence of metal ions.

**Figure 2:**
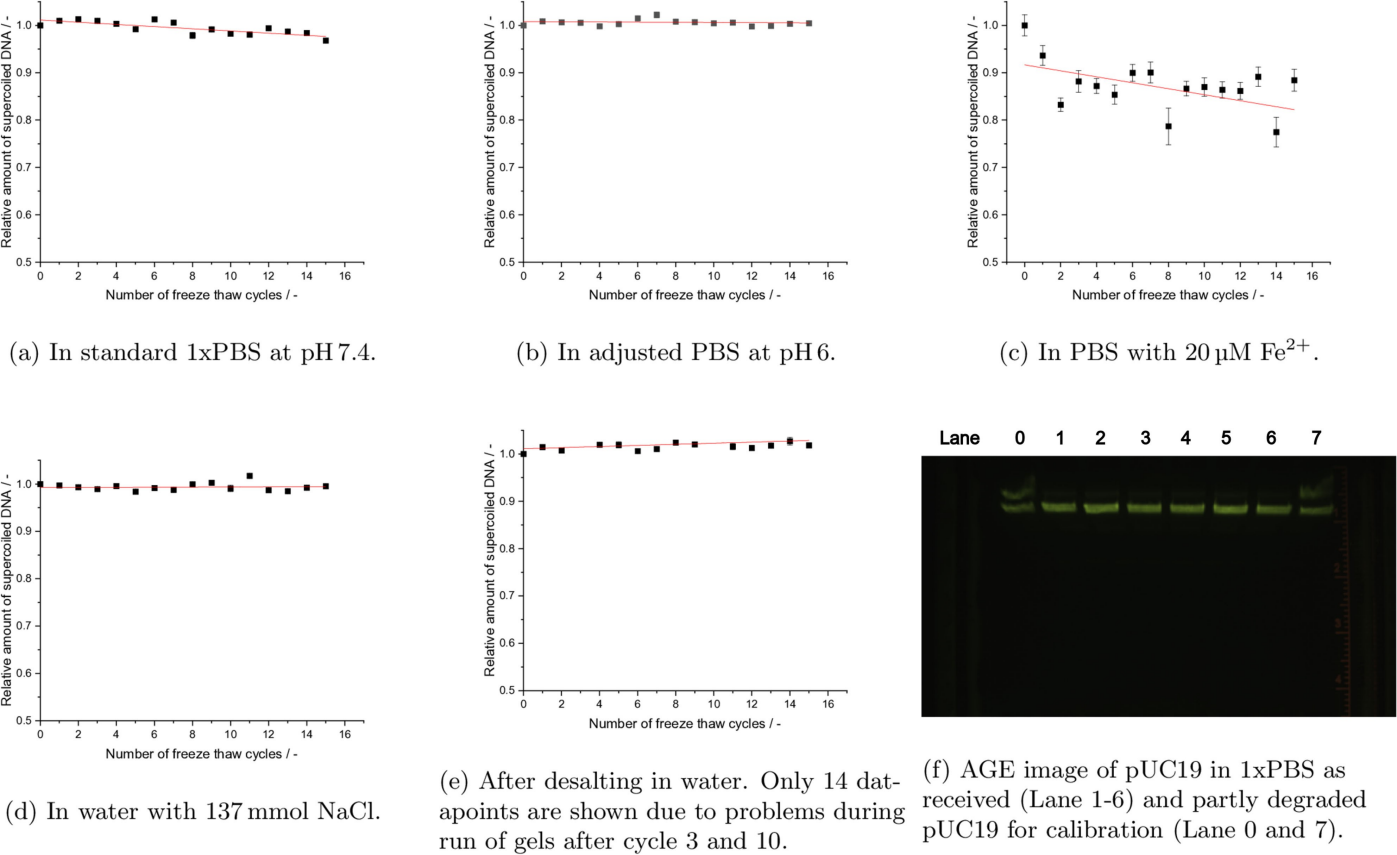
Effect of multiple slow freeze‐thaw cycles on DNA in different solvent conditions, each point is an average over n=8 measurements, error bars represent standard error of the mean (SE). When error bars are invisible, they are smaller than the respective symbol used for plotting. Red lines are fits by linear regression. For details on buffer conditions compare the text.

**Figure 3:**
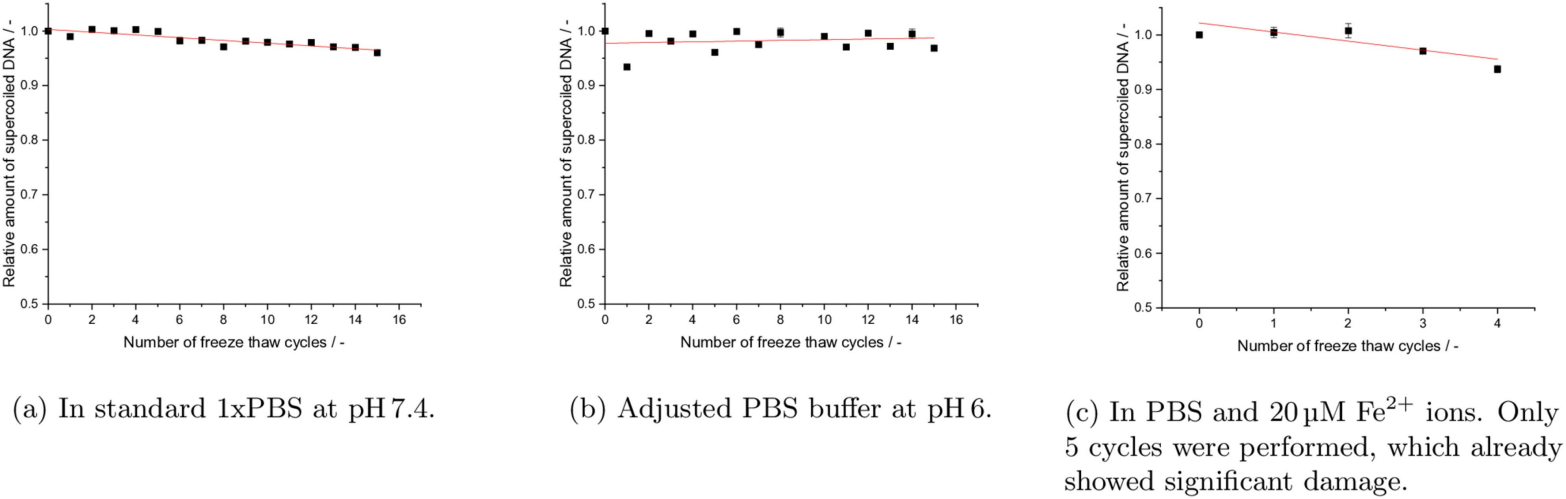
Effect of quick freezing on DNA in PBS to −196 °C in liquid nitrogen and then quick rethawing it at 50 °C. Detail in caption of Figure 2.

With respect to the first mechanism, mechanical stress, different experiments under well controlled pH and in high‐purity buffer (1xPBS) were performed. The specific mechanical stress caused by repeated freeze‐thaw cycles under slow (Figure 2a) and fast (Figure 3a) conditions, did only show very weak damage induction over 16 cycles.

Here, it is worth noting that mechanical stress can not only occur during freezing or thawing, but as well through other sample‐handling conditions, such as vortexing.^[36]^ However, for small plasmids (<5 kb) Levy *et al*. stated, that these effects do not play a significant role in the degradation of the DNA, while they become significant for DNA longer than 20 kb.^[37]^ Similarly Xin *et al*. tested the effects of repeated freeze‐thaw cycles on DNA in the context of DNA‐origami, and found a size dependent increase in sensitivity of the DNA, while protective effects by the cryoprotectants glycerol and trehalose were observed, which is in agreement with the result further discussed below.^[18]^ To test the validity of these results for the highly supercoiled plasmid DNA pUC19 with 2686 bp, we exposed our samples to repeated cycles of mechanical stress by vortexing, as it often is performed in routine workflows. Here, no degradation could be detected (data not shown). Furthermore, the damage induction was greater in the quick freeze‐thaw experiments than in the slow freeze‐thaw experiments (Table 1). Usually, slow‐freezing in a freezer allows a longer time for the rearrangement of water molecules compare to the much quicker processes when samples are immersed in liquid nitrogen. This rearrangement can lead to stronger shear forces on the biomolecules. Therefore quick‐freeze methods to obtain vitreous ice are routinely applied in cryo‐electron‐microscopy, where structure preservation of the molecules is of uttermost importance.^[38]^ Thus, the observation of higher damage in the quick freeze‐thaw experiments compared to the slow freeze‐thaw experiments, makes a dependence on the temperature during the thawing process likely, which in turn indicates that the damage processes are of chemical nature (hypothesis 2. or 3., pH or ROS mediated, respectively) and not purely mechanical (hypothesis 1.), where one would expect opposite effects.

**Table 1:** Summary of the experimental conditions and their results from linear fiting. Here, a negative slope represents a measure of the degradation per cycle and time, respectively. For details see the text.

Buffer condition	Treatment	Slope	Unit^−1^	Figure	Slope ≠0
1xPBS at pH 7.4	Slow freezing and thawing	-0.0023±0.0006	cycle	2a	yes
Adjusted PBS at pH 6	Slow freezing and thawing	-0.0002±0.0005	cycle	2b	no
1xPBS at pH 7.4 and FeCl_2_	Slow freezing and thawing	-0.007±0.003	cycle	2c	yes
Water+physiological NaCl	Slow freezing and thawing	0.0001±0.0005	cycle	2d	no
Millipore Water	Slow freezing and thawing	0.0011±0.0009	cycle	2e	no
1xPBS at pH 7.4	Quick freezing and thawing	-0.0025±0.0004	cycle	3a	yes
Adjusted PBS at pH 6	Quick freezing and thawing	0.001±0.002	cycle	3b	no
1xPBS at pH 7.4 and FeCl_2_	Quick freezing and thawing	-0.017±0.008	cycle	3c	yes
1xPBS at pH 7.4	Long term storage below −25 °C	-0.0018±0.0004	month	5a	no
1xPBS at pH 7.4	Short term storage at 4 °C	-0.0034±0.0008	week	5b	yes
1xPBS at pH 7.4	Vortexing	-0.0001±0.0004	cycle	–	no

The second mechanism, ascribed to acidic pH, was tested for various conditions. According to the literature[^32^, ^33^] acids can catalyze depurination of DNA. Thus, acidic pH can induce DNA degradation by a two‐step process, starting with depurination and a subsequent *β*‐elimination, leading to the cleavage of the sugar‐phosphate backbone.[^4^, ^33^] Hereby it is worth noting, that the rate of depurination is four‐fold higher in ssDNA compared to dsDNA.^[33]^ Therefore, it is assumed, that working with buffers in acidic pH, or with unbuffered solutions, such as ultrapure water, might influence DNA stability negatively. To test this model, DNA was prepared in Millipore water (Figure 2e), and in water with additional physiological sodium chloride concentrations (Figure 2d). Usually, pure water or sodium chloride solutions are expected to have a neutral pH. Still, under standard laboratory conditions the pH of these solutions can vary and become slightly acidic by the inevitable process of dissolving ambient carbon dioxide. However, the repeated freeze‐thaw cycles did not reveal any degradation, neither in desalted water (Figure 2e), nor in unbuffered salt solution (Figure 2d). In a second step, the effect of well defined acidic pH in a buffered solution was tested by repeated freeze‐thaw cycles under slow (Figure 2 b) and fast (Figure 3b) freezing and thawing, as described above. Here, no damage induction over the 16 freeze‐thaw cycles was observed for any of these conditions. From these combined results for DNA undergoing slow and quick freeze‐thaw cycles in PBS at pH 7.4, NaCl solutions, ultrapure water and conditions with pH 6, it can be concluded, that mechanical stress alone, as assumed in the first hypothesis, does not seem to affect 2686 bp long plasmid DNA. Furthermore, we can conclude with respect to the second hypothesis, that pH itself does not add an additional detrimental multiplicative effect on the stability of plasmid DNA during the freeze‐thawing cycles. On the other hand, very weak damage was observed in PBS buffer, independently of the slow and quick freeze‐thawing velocities, which was not observed for the other buffer conditions discussed so far. This might indicate impurities or specific mechanisms related to phosphate containing buffers, which is discussed further below, in the context of Fe^2+^ containing solutions.

**Figure 4:**
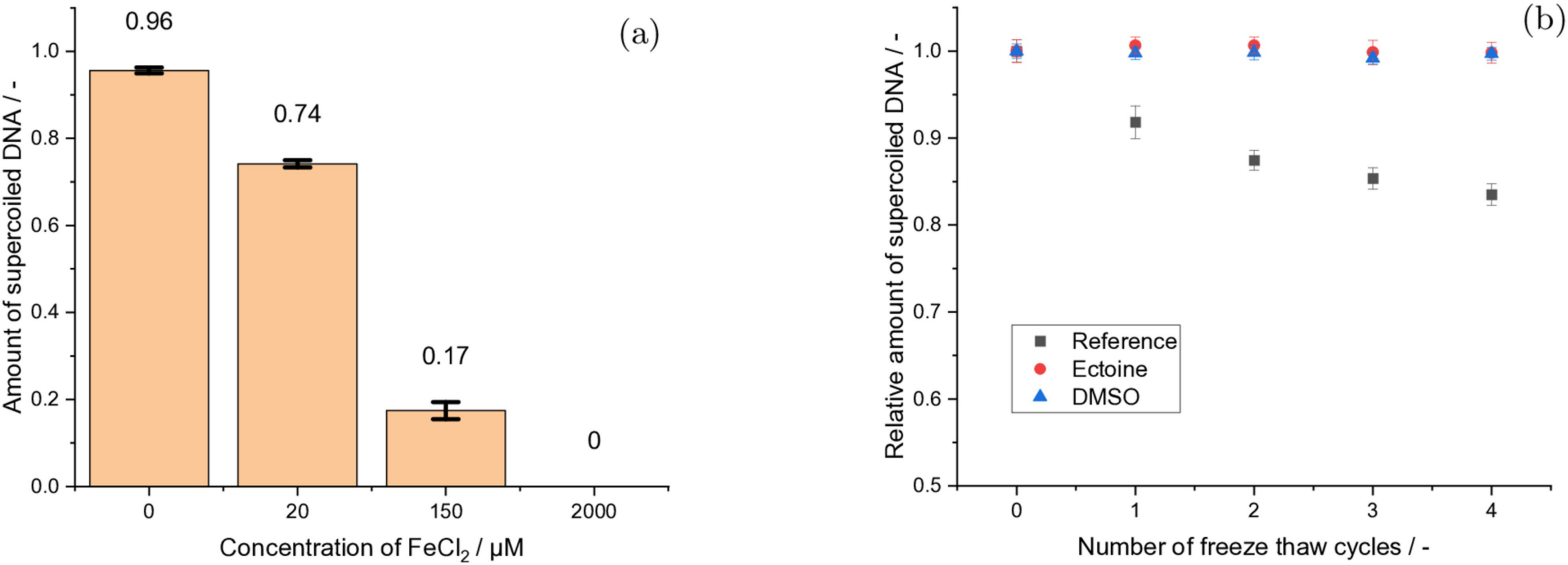
Damage induction under the presence of of iron ions and ROS. (a) Absolute amount of supercoiled DNA after addition of FeCl_2_. X‐axis represents FeCl_2_ concentrations. Each point is an average over n=8 measurements, error bars represent the standard deviation. (b) Effect of quick freezing on DNA under the presence of FeCl_2_, either without scavengers (black squares) or with the scavenger Ectoine (red circles) or DMSO (blue triangles) in 1xPBS at pH 7.4. Errorbars represent SE.

The third hypothesis assumed oxidative damage from ROS, which can be, for example, produced by Fenton like processes under the presence of metal ions. Here, directly after the addition of Fe_2_Cl to the solution a concentration dependent damage induction was observed, even without any freeze‐thaw processes. This damage upon addition increases with increasing Fe^2+^ concentration as can be observed in Figure 4a. Additionally, in comparison to the previous tests (1. and 2.) the slow freeze‐thaw cycles of DNA in PBS under the presence of 20 μM Fe^2+^ ions, revealed a much stronger SSB induction with increasing amount of cycles (Figure 2c). An even stronger effect became visible after performing the quick freeze‐thaw experiments under the presence of 20 μM Fe^2+^ ions (Figure 3c). In comparison to pure PBS the damage per cycle was about three and six times higher under slow and quick freeze‐thaw conditions, respectively (Table 1). Therefore, it can be assumed, following the third hypothesis, that metal ions contribute strongest to the degradation of the DNA samples during freeze‐thaw processes. This is most likely due to oxidative damage caused by ROS catalyzed by their presence.^[34]^ Therefore, an additional question arises immediately: What could be the underlying physico‐chemical reasons for the additional ROS production when the system is undergoing a phase‐transition in the presence of Fe^2+^? Usually, frozen states are associated with less reactivity and the rate of autoxidation of Fe^2+^ is considered to be slow.^[39]^ However, freezing or thawing processes in multicomponent systems can lead to a stark variation in local concentrations and alter reaction mechanisms and rate constants.[^40^, ^41^] This can happen as long as the system is above its eutectic point.^[40]^ Additionally, the fact, that phosphate can catalyze Fe^2+^ oxidation due to its preference for Fe^3+^, $(Fe^{2+}+O_{2}->Fe^{3+}+O_{2}^{.-}$ ) might provide a mechanistic explanation for ROS production even in the absence of H_2_O_2_.^[42]^ This is of special importance when we consider that *via* an additional step OH radicals can be produced, even without the presence of H_2_O_2_.^[42]^ Thus, ROS can break covalent‐bonds within DNA, which implies not only damage at the sugar‐phopsphate backbone, but as well damage to the N‐glycosidic‐bond between deoxyribose and nucleobases.^[22]^ This breakage of the N‐glycosidic‐bond can lead to a base loss and therefore loss of information when DNA is used as a molecule to encode information.^[43]^ Here, especially the highly reactive hydroxyl radical is very efficient in causing SSB and base damage in DNA.^[34]^ To provide further evidence and to test these mechanisms, additional experiments with the radical scavenger Ectoine^[23]^ and DMSO^[34]^ were performed. The presence of both cosolutes showed nearly a complete suppression of the damage during repeated quick freeze‐thaw cycles (Figure 2b, DMSO: blue triangle, Ectoine: red circle) despite the presence of Fe^2+^. This is remarkably, since the quick freeze‐thaw experiments without scavenger molecules, showed the strongest damage induction of all freeze‐thaw conditions. Here we note, that both scavengers have various biotechnological applications and are with respect to a range of properties rather different. For example, DMSO is assumed to interfere with some cellular processes,^[44]^ while Ectoine is termed a compatible solute, which can be accumulated by cells in molar concentrations, without disturbing their metabolism.[^24^, ^45^] DMSO can cause denaturation of proteins.,^[46]^ while Ectoine is a kosmotrop.^[47]^ Furthermore, the zwitterion and osmolyte Ectoine tends to accumulate at the DNA phosphate backbone^[24]^ and to influence the water structure in its surrounding,[^45^, ^47^] and therefore might be providing additional damage mitigating effects similar to other cryoprotectants such as glycerol or trehalose besides the actual scavenging.^[18]^ However, when we invoke Occam's razor, it is reasonable to assume, that the protection of DNA by Ectoine and DMSO is most likely based on their common property, the scavenging of ROS. The study of their detailed scavenging reactions is of interest for further applications in pharmacy or DNA based data storage, but beyond the scope of this study. Other common cryoprotectants which possess scavenging capabilities, such as glycerol or trehalose, are expected to provide protection against these degradation pathways as well.

**Figure 5:**
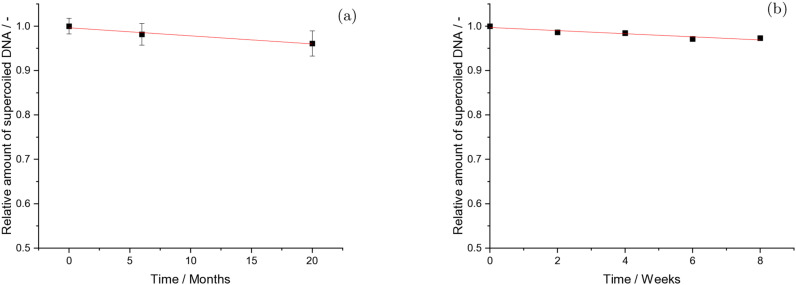
(a) Time dependent amount of undamaged DNA during storage at T≤−25 °C Each datapoint represents the average of ten independent samples and the errorbars their SE. Each average of theses samples was determined from six replicates. (b) T=4–5 °C in 1xPBS at pH 7.4. Each datapoint is an average over n=8 measurements from one sample stored for in total eight weeks. The error bars represent the SE.

In the light of the results involving the addition of scavenger, it seems to be likely, that the damage which was observed in the presence of standard 1xPBS buffer, as received from the manufacturer, is similarly related to the presence of trace metals with concentrations far below 20 μM. Since the presented results indicate, that the damage is not caused by the metals ions themselves, but by radicals formed by them, it is reasonable to assume that the findings about Fe^2+^ are likely to be valid for other metal ions (*e. g*. Co^2+^, Ni^2+^, Cu^+^, compare^[48]^), which undergo Fenton‐type or Haber‐Weiss reactions. Furthermore, it can be assumed that Tris (Tris(hydroxymethyl)aminomethan) based buffers, commonly used in various biochemical applications, provide some intrinsic against the observed ROS based degradation mechanisms due to the fact, that Tris can act as a radical scavenger.^[34]^ Since the question about the detrimental effects of freeze‐thaw cycles on DNA originated from the discussion about the advantages and disadvantages of different storage conditions, it is of importance to discuss the related stability of DNA under short and long term storage. Short term storage of plasmid DNA for two weeks at temperatures around 4–5 °C, in 1xPBS did cause very little degradation (Figure 5b), and can therefore be considered as a possible alternative to freezing, when other substances, such as proteins, are present in a formulation, which might show a higher susceptibility to repeated free‐thaw cycles than DNA. However, this should be only considered if freezing is not an option for the reasons mentioned above. Because above T>0 °C the efficiency of these detrimental processes depend on the complex interplay of various parameters such as temperature, ionic strength, pH and DNA structure and have to be evaluated carefully in advance.[^4^, ^33^]

Due to the time consuming nature of long‐term studies, very few were performed in the past. Among them, one study investigated the long term stability of plasmid DNA pCMVb for storage over seven years at −20 °C (150 mM Na_2_HPO_4_ NaH_2_PO_4_ buffer, pH 7.0). There, no decrease in the relative amount of plasmids in the supercoiled form was observed.^[49]^ Similar results were obtained for storage of plasmid DNA pCMVb for up to 13 months at −80 °C.^[5]^ However these previous studies used a small sample size and were lacking a detailed statistical analysis. Therefore our results describe for the first time the topological long‐term stability of highly supercoiled plasmid DNA under well controlled conditions. Here, the degradation at temperatures below −25 °C proceeds much slower, on the order of years (Figure 5a). In the linear approximation, the slope of the long‐term stability curve resulted as $\left(-0.18\pm 0.04\right)\;$ % month^−1^. Setting this value into relation with the slope for short‐term storage at 4 °C: $\left(-0.34\pm 0.04\right)\;$ % week^−1^ and the value originating from slow freeze‐thawing of plasmid DNA in 1xPBS as $\left(-0.23\pm 0.06\right)\;$ % cycle^−1^ one can estimate the optimal procedures for individual applications to minimize damage induction during handling and storage of DNA.

## 3 Conclusion

With respect to the different applications of DNA the amount and type of damage has varying importance. In DNA data storage DNA damage can lead to complete loss of information when a part of a DNA molecule is abstracted, which can, for example, happen after multiple strand‐breaks.^[1]^ Furthermore, loss of bases could possibly lead to data corruption. However this is less likely when only single bases are of concern, due to the duplex structure of the DNA molecule and the additional encoding in the complementary base. Here, nature already provides a natural way of error correction. For DNA based biodosimetry[^8^, ^10^] the structural, macroscopic transformation of plasmid DNA upon the induction of only one SSB enables the high sensitivity of these “molecular dosimeters”. Additionally, complex types of damages, such as double‐strand breaks (DSB), can be distinguished and quantified with ease.^[50]^ The sensitivity for damage detection is highest, when initially the amount of undamaged DNA within the ensemble is as high as possible (*e. g*. above 90 %). Therefore, the result presented here, provide a guideline for selecting the optimal conditions to avoid non‐radiation induced DNA damage during biodosimetric experiments. In quality control, the induction of a SSB provides the similar advantages in terms of sensitivity or the detection of modifications of chemical bonds by ROS or enzymes, as discussed in terms of dosimetric applications. For pharmaceutical applications of plasmid DNA the DNA has to remain stable for extended periods, whereby the trade off between long term stability at ambient temperature and freeze thaw effects has to be considered. However, compared to protein based drugs, DNA can be considered more stable, since it is comparatively less dependent onto the integrity of their higher order structure.^[3]^ Thus, plasmid DNA requires much more fundamental modifications to loose its functions. Hereby breakage of covalent bonds at the sugar‐phosphate backbone or the glycosidic bond between DNA base and sugar are, together with intra‐molecular and inter‐molecular crosslinking of DNA, prime examples.

In conclusion, all these applications have in common, that keeping the contamination by trace metals at the minimum possible level is of uttermost importance, while the effect of mechanical stress on plasmid DNA is much lower than previously thought.

## Experimental Section

### Plasmid DNA

Double‐stranded DNA (dsDNA), pUC19 plasmid DNA was obtained from Plasmidfactory (Bielefeld, Germany) at a concentration of 500 ng μL^−1^ in 1xPBS. The PBS buffer (Plasmidfactory, Bielefeld, Germany) has a physiological pH of 7.4 and consists of Millipore water with 137 mmol L^−1^ NaCl, 2.7 mmol L^−1^ KCl, 10 mmol L^−1^, Na_2_HPO_4_ and 1.8 mmol L^−1^ KH_2_PO_4_. Additionally, plasmid DNA pUC19 at a concentration of 1 μg μL^−1^ with 137 mmol NaCl (Sigma, Suprapur with a purity of 99.99 %) was purchased from Plasmidfactory (Bielefeld, Germany). These NaCl concentrations correspond to the concentration as in 1xPBS. The DNA samples in Millipore water (pH 7.5) were prepared using dialisys by a Float‐a‐lyzer G2 (with a molecular weight cutoff of 8–10 kDa, Spectrum Labs) following the manufacturers instructions. Such a dialisys procedure removes most of the salts from the solution. However, some Mg^2+^ can be extected to persist as counterion at the DNA sugar‐phosphate backbone.^[51]^

### Agarose gel electrophoresis

**Figure 6:**
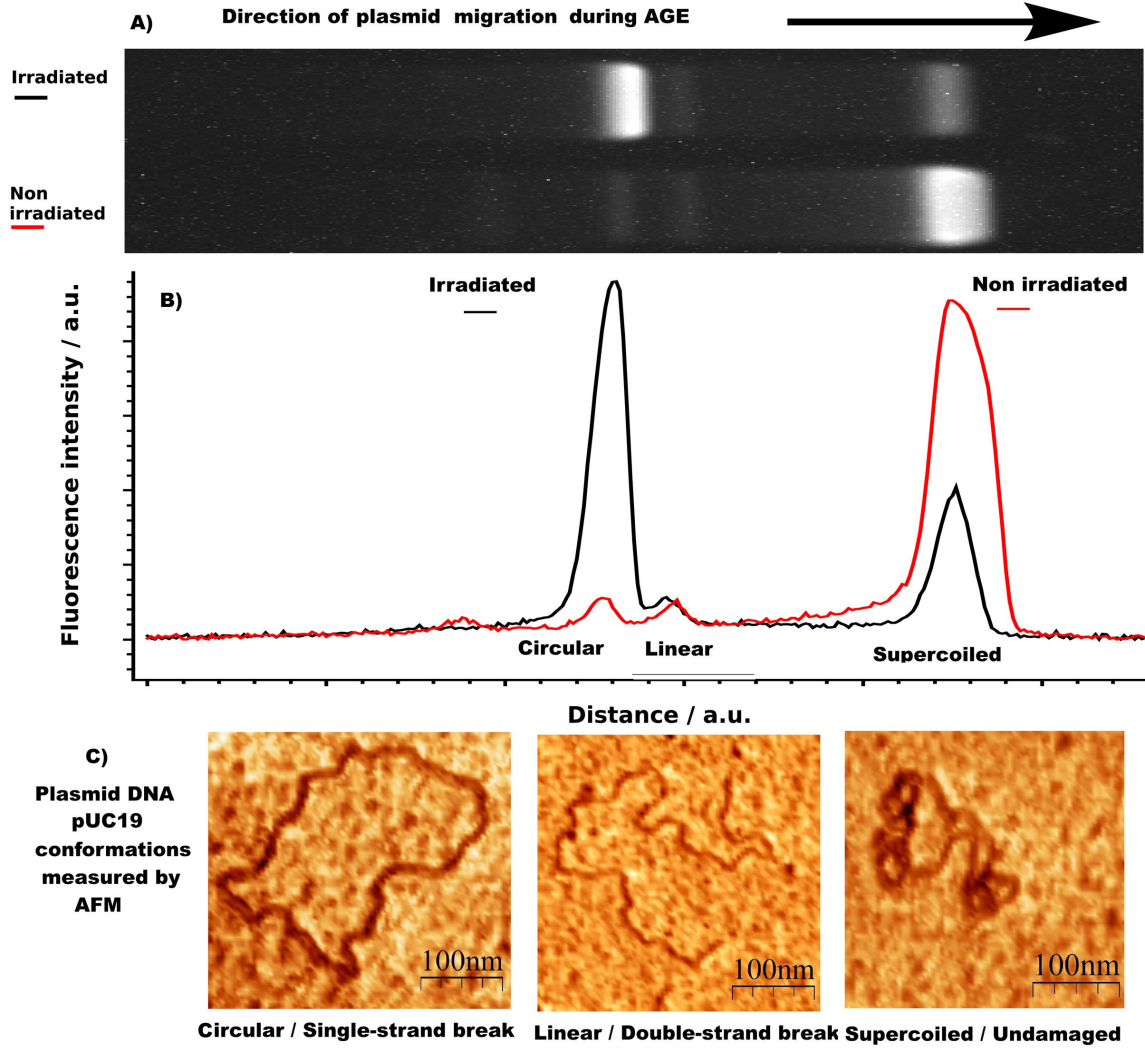
Example of DNA damage determination by agarose‐gel electrophoresis a) Two gel lanes of irradiated and non‐irradiated plasmid DNA pUC19 of various conformations. b) Associated fluorescence intensity extracted from the images shown above. c) Exemplary images of different plasmid DNA conformations measured by atomic‐force microscopy: (Left) Circular DNA with one single‐strand break. (Center) Linear DNA with a double‐strand break. (Right) Supercoiled, undamaged plasmid DNA pUC19.^[52]^ Image taken and modified with permission from [53].

Agarose gel electrophoresis (AGE) was performed to determine the damage to the DNA samples (Figure 6a). The measurements were performed using 1 % agarose gels and 1xTAE buffer at 5 V cm^−1^ for either 30 or 60 min per measurement. For each gel 8 channels were loaded with 2 μL of DNA and pre‐stained using 8 μL gel loading buffer with 50xSyBr Gold (Thermo) each. The DNA damaged was quantified by measuring the fluorescence of the different conformations of DNA in each lane and averaging over 8 samples for each measurement.

### Quantification of DNA damage and stability

The stability of DNA is related to the integrity of the DNA sugar‐phosphate backbone, as well as the intactness of the DNA bases and their sequence. In supercoiled (SC) plasmid DNA the degradation or instability of the DNA sugar‐phosphate backbone is linked to the conversation of the topological constrained form of the SC plasmid DNA to the open‐circular (OC) form. Therefore, undamaged plasmid DNA is the ideal model system to study DNA stability and degradation, since it exists in a covalently‐closed circular/SC form (Figure 6c right), which is topologically constrained. When a single‐strand‐break (SSB) at the sugar‐phosphate backbone occurs in such a plasmid, it relaxes to the OC form (Figure 6c left). In the case of a double‐strand‐break (DSB) it changes even further, from the OC form to a linear conformation (Figure 6c center). Thus, this “macroscopic” property, naemly the topological isoform of the plasmid, is intrinsically linked to the “microscopic” intactness of the covalent bonds within the sugar‐phosphate backbone of the DNA molecule. Therefore the applied methodology provides information about the occurrence of covalent‐bond breakage with high senistivity within DNA which is othwerwise complicated to achieve in a quantitative manner for a huge ensemble of molecules.^[8]^ These three states, undamaged DNA (SC form), DNA with a SSB (OC‐form) or a DSB (linear form), were quantified by AGE. The intensity profiles of the gels (Figure 6b) were then analyzed by subtraction of a linear background and integration over the peaks corresponding to the supercoiled and damaged DNA respectively. The attachment efficiency of SYBR Gold to supercoiled DNA compared to the open circular form is 1.05, therefore no correction of the intensities values of the different conformations is needed.^[23]^

### Quantitative analysis

The amount of the undamaged plasmid DNA in dependence of a single damage event is best described by an experimental decay,^[8]^ which can be approximated in the low damage regime by a linear stress‐damage dependence.^[50]^ Therefore damage was analyzed and compared by a linear regression after the initial datapoint was used for normalization. Slopes and uncertainties of this linear regression (Origin 2020) were summarized in Table 1. Hereby a negative slope indicates a decrease of the relative amount of the SC form of the plasmid DNA which is linked to induction of SSB. The data was tested against the hypothesis (p‐value 0.05) that the slope is significant different from zero.

### Freeze‐thaw experiments

The DNA for the freeze‐thaw experiments was stored at T<−20 °C until the experiments. When the DNA was thawed for the first time the relative amount of supercoiled DNA and damaged DNA respectively were determined using AGE. This first value was used for normalization in all graphs were relative amounts of supercoiled DNA are shown. Concentration and purity were determined by UV‐Vis absorption at 260 nm and 280 nm. Then the samples were frozen over night at T<−20 °C and slowly rethawed at 4 °C, the samples were homogonized by vortexing for 3 s at 10 Hz, the relative amount of supercoiled DNA was determined using AGE and the samples were frozen again. This was repeated for 15 cycles in total.

### pH dependence

One sample of 500 ng μL^−1^ pUC19 in 1xPBS (140 mM NaCl, 2.7 mM KCl, 10 mM Na_2_HPO_4_) was adjusted to pH 6 using 1 M HCl. The relative amount of supercoiled DNA in the sample was determined using AGE. Then the sample was frozen at T<−20 °C and then slowly thawed at 4 °C. The relative amount of supercoiled DNA was determined again and the experiment repeated for a total of 15 freeze‐thaw‐cycles. To test the pH stability of the unbuffered solutions a sample of 500 mL of deionized water (pH 7.5) was put under an atmosphere of 95 % N_2_ and 5 % CO_2_ at 1 bar without bubbling the gases through the sample. The sample was left under this atmosphere for 1 h at room temperature before the pH was determined again, resulting in a slight decrease of pH 6.5.

### Metal ions

To investigate the influence of traces of metal ions a sample of 300 μL of 500 ng μL^−1^ plasmid DNA in 1xPBS was prepared. To this sample 3 μL of a 2 mM FeCl_2_ (Merck) solution was added for a final concentration of Fe^2+^ of 20 μM. Similarly, the isolated effects of addition of higher concentrations (final values: 20 μM, 150 μm, 2000 μm) of FeCl_2_ without the involvement of freeze‐thawing processes were tested. Here, the AGE analysis was performed 30 min after the addition of FeCl_2_ stock solutions to separate “addition” and freeze‐thaw effects.

### Accelerated freeze‐thaw experiments

Additionally, three samples (pure, pH 6, with 20 μM Fe^2+^) of pUC19 DNA in PBS (500 ng μL^−1^) were prepared as described above. The relative amount of supercoiled DNA of all samples was determined using agarose gel electrophoresis. Then the samples were submerged in liquid nitrogen at −196 °C for 10 minutes and then immediately rethawed at 50 °C for 10 minutes. The relative amount of supercoiled DNA was determined again and the experiment repeated for a total of 15 times.

### Test of the involvement of ROS in damage induction under the presence of iron ions

Additional experiments under the presence of metal ions, namely Fe^2+^, were repeated in PBS at pH 7.4 to test the proposed damage mechanisms related to ROS. Therefore some experiments were repeated under the presence of the hydroxyl radical scavenger dimethyl sulfoxide (DMSO) as well as the radical scavenger and compatible solute Ectoine (1,4,5,6‐tetrahydro‐2‐methyl‐4‐pyrimidinecarboxylic acid) both obtained from Sigma. Afterwards the amount of undamaged plasmid DNA was determined before FeCl_2_ was added to a final concentration of 20 μM. Then the amount of damaged of undamaged plasmid DNA was determined immediately and the samples were subjected to repeated quick freeze‐thaw experiments as described above. To avoid any influence of purely time dependent effects, the samples handling times in the non‐frozen state were held as short as possible and AGE was performed immediately after thawing.

### Long‐term storage at T<−25 °C

To test the long term stability, an extensive set of the plasmid pUC19 samples was stored in aliquots of 500 ng μL^−1^ in sterile cryotubes within 1xPBS in the dark at temperatures below T≤-25 °C. At the beginning of the study, ten samples were selected randomly and analyzed as described above. After six and twenty month, this analysis was repeated for ten independent and randomly chosen samples from the same batch.

### Short‐term storage at T=4–5 °C

To test the short term stability, DNA in 1xPBS was stored at 4 °C in presence and absence of 150 μM FeCl_2_ for up to 8 weeks. The relative amount of supercoiled DNA of all samples was determined at the beginning of the experiment using AGE and every two weeks after that for a total of 8 weeks.

### Mechanical stress by sample handling

To determine the damage caused by the mechanical stresses during common sample handling techniques such as pipetting and vortexing the DNA samples were subjected to the following treatment: A sample of pUC19 plasmid DNA in PBS was vortexed at 10 Hz for 10 s, then the damage was quantified using AGE and the vortexing repeated for a total of 16 times.

## Conflict of interest

The authors declare no conflict of interest.

4

## Data Availability

The data that support the findings of this study are available from the corresponding author upon reasonable request.
